# Medical and Dental Applications of Titania Nanoparticles: An Overview

**DOI:** 10.3390/nano12203670

**Published:** 2022-10-19

**Authors:** Afsheen Mansoor, Zohaib Khurshid, Muhammad Talal Khan, Emaan Mansoor, Faaz Ahmad Butt, Asif Jamal, Paulo J. Palma

**Affiliations:** 1 Department of Dental Material Sciences, School of Dentistry, Shaheed Zulfiqar Ali Bhutto Medical University, Islamabad 44080, Pakistan; 2 Department of Prosthodontics and Dental Implantology, College of Dentistry, King Faisal University, Al-Ahsa 31982, Saudi Arabia; zsultan@kfu.edu.sa; 3 Department of Dental Biomaterials, Bakhtawar Amin Medical and Dental College, Multan 60650, Pakistan; tkhaan747@gmail.com; 4 Islamic International Dental College, Riphah International University, Islamabad 44000, Pakistan; emaanmansoor5@gmail.com; 5 Department of Materials Engineering, NED University of Engineering & Technology, Karachi 74200, Pakistan; faazbutt@cloud.neduet.edu.pk; 6 Department of Microbiology, Quaid-i-Azam University, Islamabad 45320, Pakistan; asifjamal@qau.edu.pk; 7 Center for Innovation and Research in Oral Sciences (CIROS), Faculty of Medicine, University of Coimbra, 3000-075 Coimbra, Portugal; 8 Institute of Endodontics, Faculty of Medicine, University of Coimbra, 3000-075 Coimbra, Portugal

**Keywords:** nanomaterials, nanoparticles (NPs), nanodentistry, medicine, and titanium oxide (TiO_2_)

## Abstract

Currently, titanium oxide (TiO_2_) nanoparticles are successfully employed in human food, drugs, cosmetics, advanced medicine, and dentistry because of their non-cytotoxic, non-allergic, and bio-compatible nature when used in direct close contact with the human body. These NPs are the most versatile oxides as a result of their acceptable chemical stability, lower cost, strong oxidation properties, high refractive index, and enhanced aesthetics. These NPs are fabricated by conventional (physical and chemical) methods and the latest biological methods (biological, green, and biological derivatives), with their advantages and disadvantages in this epoch. The significance of TiO_2_ NPs as a medical material includes drug delivery release, cancer therapy, orthopedic implants, biosensors, instruments, and devices, whereas their significance as a dental biomaterial involves dentifrices, oral antibacterial disinfectants, whitening agents, and adhesives. In addition, TiO_2_ NPs play an important role in orthodontics (wires and brackets), endodontics (sealers and obturating materials), maxillofacial surgeries (implants and bone plates), prosthodontics (veneers, crowns, bridges, and acrylic resin dentures), and restorative dentistry (GIC and composites).

## 1. Introduction

The advent of nanotechnology has led to a substantial surge in the applications of NPs for biomaterials, antibacterial substances, drug delivery systems, electronic equipment, sunscreens, and cosmetics [1,2]. Nanomaterials have previously been defined as natural, manufactured, or incidental materials that include particles as agglomerates or aggregates or in an unconfined form where 50% or a higher number of the particles depict one or more outer dimensions within the range of 1–100 nm [3]. Moreover, to consider a material as a nanoparticle one of its three dimensions should be in a range of 1–100 nm [4,5]. Unique chemical and physical properties are demonstrated by oxide NPs due to their limited size and their corner surface sites possessing higher density [6]. The physicochemical properties of fine particles (FPs) vary as compared to the NPs (NPs) that have a similar composition. 

The TiO_2_ fine particles (FPs) are poorly soluble and have a low toxicity [7,8]. The TiO_2_ NPs (NPs) have an enhanced catalytic activity as compared to the fine particles (FPs), which consequently leads to their higher utilization in consumer and industrial products. The properties of the TiO_2_ NPs may pose a challenge to human health and are responsible for the distinctive bioactivity of these NPs [9,10]. The TiO_2_ NPs are prominent and versatile oxides that have a higher production due to their acceptable chemical stability, lower cost, strong oxidation properties, high refractive index, and formidable oxidation properties [11]. The TiO_2_ NPs have found their optical industrial applications as semiconductors due to having the critical property of the wide band gap [12,13]. They have also been used in electronic devices and as sensors due to their particular ionic and electrical properties [14]. As these NPs exist as a water insoluble powder and are white in color, they can be utilized in the paint industries as pigments [15]. Three polymorphic forms of the TiO_2_ NPs exist naturally; they are the rutile, brookite, and anatase forms with crystalline structures, and the gemstone industries have utilized them extensively [14]. The TiO_2_ NPs have been employed for decomposing water waste pollutants as photocatalysts, as food additives, and as antibacterial and antimicrobial agents [16,17,18,19]. The TiO_2_ NPs can help in destroying fungi, viruses, bacteria, and cancer cells [20]. The TiO_2_ NPs have been used in sonodynamic and photodynamic therapy (PDT) for the treatment of cancer [21]. The NPs have the ability to convert photon energy into heat through photo thermal therapy, via their super hydrophilicity, in vivo chemical and thermal stability, low toxicity, optical absorption, and thermal conductivity [22]. The nanostructure of TiO_2_ has been used for drug delivery systems in various anticancer drugs, such as temozolomide, cisplatin, doxorubicin, and daunorubicin [23,24,25]. Previously, polyethylene glycol-coated NPs have been used for the treatment of melanoma through photothermal therapy [26]. The (TiO_2_) NPs have been used in dental applications due to their ability to enhance the mechanical properties of biomaterials without altering their biocompatibility [27,28,29]. Previous studies have utilized the TiO_2_ NPs in order to improve the bond strength and antimicrobial properties of composites [30]. The TiO_2_ NPs have also been used to improve the working time, setting time, push-out bond strength, and compressive strength of cements [31]. The mechanical properties of the resins have also been optimized by incorporating titania NPs into them [32,33]. Moreover, silver-doped titania NPs have also been incorporated into dental polymers in order to produce potent bactericidal effects [34].

## 2. Background of Nanotechnology

Nanotechnology has been considered as a significant discipline of the latest research involving the synthesis protocols and the designing and manipulating techniques of the particle’s structure in different dimensions in the range of about 1–100 nm. Moreover, this emerging nanotechnology has specifically opened vast doors for the synthesis of nano-scaled materials and the exploration of their unique electronic, optical, physicochemical, strength, magnetic, electrical, and mechanical characteristics, together with the utilization of this rapidly growing novel technology in certain various fundamental frontiers. These fundamental areas that involve nanotechnology are electronics, multimedia, cosmetics, medicine, engineering, electronics, industries, food, environment, robotics, optics, machines, catalysts, drug delivery systems, genomics, biomedicines, material sciences, energy sciences, light emitters, solar energy processes, imaging technology, physics, chemistry, electromagnetic devices, electron transistors, energy-producing plants, water treatment plants, solar energy conversion, etc. [35,36]. Currently, nanotechnology has gained a popular status in multiple health systems, such as control, prevention, diagnosis, monitoring, and treatment; this has happened quite rapidly because of its unique and high-quality properties [37,38]. The word “nano” is taken from the Greek word nanos, meaning “dwarf”, which factually refers to 1 billionth of physical size. Furthermore, it can be inferred that the area of nanotechnology works at the level of atoms and molecules ranging between 1 and 100 nm in size [39]. 

### 2.1. History of Nanotechnology

NPs were used by artisans as far back as the 9th century in Mesopotamia for generating a glittering effect on the surface of pots. Pottery from the Middle Ages and the Renaissance often retains a distinct gold or copper-colored metallic luster [40]. The luster originated within the film itself, which contained silver and copper NPs dispersed homogeneously in the glassy matrix of the ceramic glaze [41]. The concepts that seeded nanotechnology were first discussed in 1959 by renowned physicist Richard Feynman in his talk “There’s Plenty of Room at the Bottom”, in which he described the possibility of synthesis via the direct manipulation of atoms. The term “nanotechnology” was first used by Norio Taniguchi in 1974, though it was not widely known at that time [38,40]. 

### 2.2. Significant Features of Nanotechnology

There are three major features that play a vital role in defining the versatility and superiority of nanotechnology in comparison to normal technology [42]:

- This technology can bring changes in the original matter’s structure by converting it into a nanoscale product.
- The newly formed nanoscale structure should contain periodic repetition (i.e., the nanoparticle must periodically repeat itself in one or more than one direction) just like the parent matter.
- The novel properties, characteristics, and functions of a newly formed nanoscale product should be like those of the parent matter or be superior to the parent matter despite being nanometric.

### 2.3. Classification of NPs

**Figure 1:**
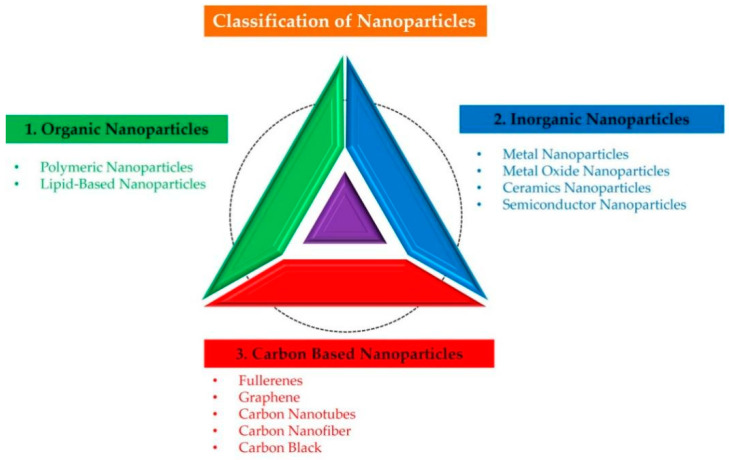
Classification of NPs: (1) organic-origin NPs, containing polymeric NPs and lipid-based NPs; (2) inorganic-origin NPs, containing metal NPs, metal oxide NPs, ceramic NPs, and semiconductor NPs; (3) carbon-origin NPs, containing fullerene NPs, graphene NPs, and carbon NPs (idea adapted from ref [45].

**Figure 2:**
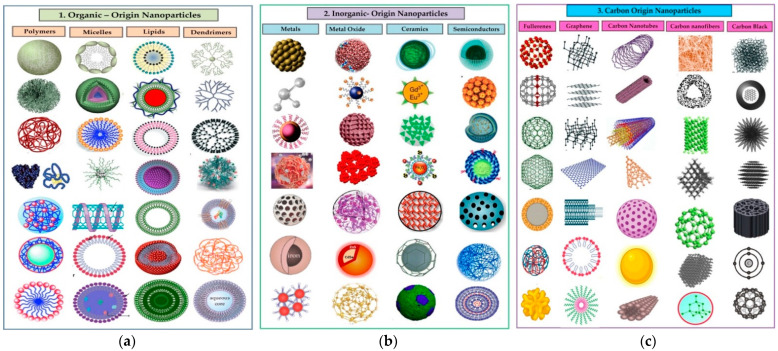
Size and shape of different types of NPs: (**a**) polymers, micelles, lipids, and dendrimers belonging to organic-origin NPs; (**b**) metals, metals oxide, ceramics, semiconductors belonging to inorganic-origin NPs; (**c**) fullerenes, copolymer, carbon nanotubes, carbon nanofibers, and carbon black belonging to carbon-origin NPs (idea adapted from the ref [46]).

NPs are categorized generally as organic, inorganic, and carbon-based in origin, as illustrated in (Figure 1). The carbon-based NPs are entirely made of carbon. The organic NPs are found to be non-toxic, biodegradable, and ideal for drug delivery. The NPs that do not consume carbon during their synthesis are called inorganic NPs and include metal and metal oxide-based NPs. The metal oxide-based NPs have extraordinary properties, such as their sizes (ranging between 10 and 100 nm); shapes (spherical, cylindrical, etc.); surface characteristics (pore size, high surface-area-to-volume ratio, surface charge dentistry, and surface charge); structures (crystalline and amorphous); color; and sensitivity to various environmental factors (heat, air sunlight, moisture, etc.) (Figure 2) [43,44].

## 3. The Titanium Oxide NPs

These NPs are commonly named as TiO_2_ or titania because they are oxides of metals [47,48,49]. Titania is available in the form of fine, white powder and/or thin film [22,50]. Naturally, there are four crystal forms of titanium oxide: TiO_2_ (B), brookite, anatase, and rutile [51]. However, the mixture of anatase and the rutile crystal phase form of the TiO_2_ NPs, which usually exists, is more inert and stable in comparison to the anatase crystal phase form, which displays more chemical reactivity. This chemical reactivity of the anatase crystal phase form could be beneficial in eliminating cancer cells efficiently [52,53].

The TiO_2_ NPs are famous for their well-known attractive properties, including magnetic, optical, thermal, physicochemical, electrical, rheological, mechanical, and biological properties. These NPs are highly suitable for utilization in various fields under normal conditions because of their relatively low cost, insoluble nature, and high refractive index (*n* = 2.4). They are absolutely extra white, which enhances their value in multiple different fields [47,48,49]. Moreover, TiO_2_ NPs are widely used in anti-flavor textiles, water disinfectants, food supplements, cosmetics, indoor sprays, anti-flavor textiles, household appliances, paper, plastics, food packaging materials, sunscreens, electronic products, medicines, fibers, bakery products, sensors, glazes, and coloring agents in foods due to their smaller size [54,55,56]. 

### 3.1. Basic Requirements for Employing TiO_2_ NPs in Medical and Dental Applications

The biocompatibility of TiO_2_ NPs in the medical and dental applications is the most important requirement of these NPs and is dependent on two main factors, which include its synthesis protocols and its characteristic features attained by characterization techniques.

#### 3.1.1. Synthesis Protocols of TiO_2_ NPs

**Figure 3:**
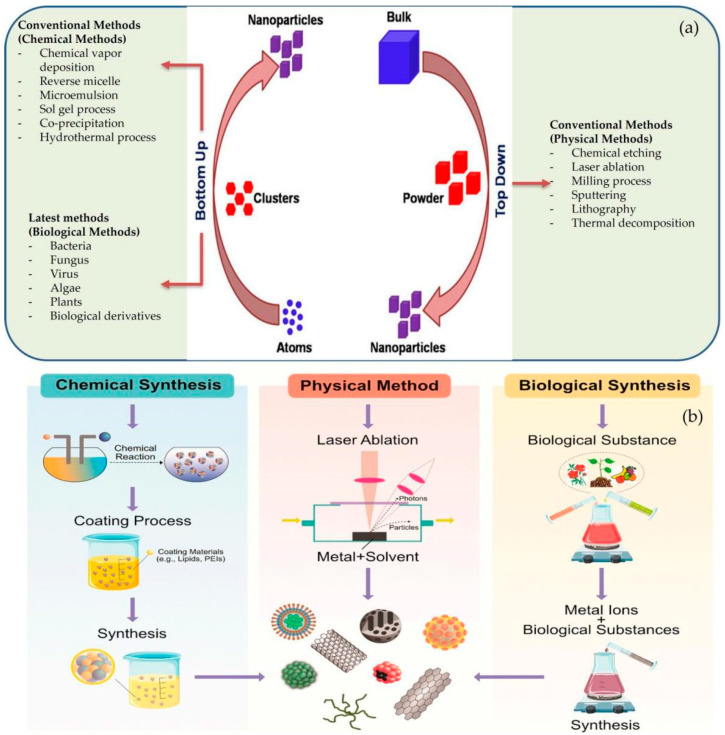
Schematic representation of protocols employed for the synthesis of TiO_2_ NPs: (**a**) chemical and physical methods involved in the conventional methods category and biological methods involved in the latest methods category, showing (**b**) chemical, physical, and biological synthesis involving both the bottom-up and top-down techniques (idea adapted from the ref [57,58].

The synthesis of NPs is carried out by two main techniques, referred to as the conventional (top-down) and biocompatible (bottom-up) methods. The physical and chemical methods of synthesis are deployed in the conventional (top-down) techniques, whereas the biological, green, and biological derivative methods are employed in the biocompatible (bottom-up) techniques to a large extent (Figure 3) [57,58]. 

##### Conventional (Top-Down) Synthesis 

The top-down technique involves the breakdown of bulk material in nano-sized particles through physical methods involving thermal decomposition, sputtering, laser ablation, nanolithography, and milling [59], whereas the chemical techniques include: co-precipitation, laser pyrolysis, the sol-gel process [60], spray pyrolysis, chemical vapor deposition [61], the aerosol process, reverse micelle [62], atomic/molecular condensation, the microemulsion process [63], and the hydrothermal method [64]. The process of the synthesis has a great impact on the transformation phase, i.e., from amorphous to anatase or rutile or brookite [65]. However, this method produces defects in the surface structure and morphology of the new material, which becomes the main limitation because the surface chemistry and physical properties of the NPs are highly dependent on the surface structure as well as the morphology [66]. The synthesis of TiO_2_ NPs via the chemical method is most widely used due to its ease of fabrication and the proper control of the size and shape of the NPs. On the other hand, its utilization is limited due to its over-expensive production, ecotoxicity, excessive energy, and non-availability of environmental sustainability, together with high temperature and pressure, which inhibit their mass production further, reducing their applications in certain different areas [67].

In addition, the electrophoretic fabrication of TiO_2_ NPs is easy as well as timesaving, but it involves the utilization of non-aqueous solvents that are harmful, as reported by Boccaccini et al. in previous research [68]. The hazardous effects, including aquatic pollution, land pollution, environmental pollution, and the destruction of wildlife as well as habitats, are caused by these non-aqueous solvents. Furthermore, these non-aqueous solvents are toxic enough to cause certain health hazards when evaporated in the surrounding air in the environment [68].

The microwave-assisted preparation of TiO_2_ NPs is widely accepted due to its time- saving capacity and its uniform, homogeneous, and quick heating of the reaction mixture at required the temperature, which is an advantage. On the other hand, this method is not cost-effective but rather expensive because it requires the increased power of heating. As this method is an energy-intensive procedure, it therefore lacks the production of TiO_2_ NPs in large amounts [69]. 

The sol-gel process of TiO_2_ NPs is extensively investigated to fabricate more crystalline NPs, followed by sol formation, gelation, and solvent removal [70]. This method is costly because it involves the utilization of expensive raw materials that lead to the cracking and volume reduction of newly formed TiO_2_ NPs after the drying process [71].

The solvothermal method employs both high pressure and temperature for longer duration (pressure < 1 atm and temperature > boiling point of water). This is a time-consuming process and requires high maintenance costs for autoclaves because the equipment used is quite expensive [72]. In addition, when additional surfactants are used in this process, they will not only make the reaction more complex but will also lead to the excessive formation of impurities [73]. 

Currently, hydrothermal methods of synthesis have been greatly employed due to their ease in fabricating TiO_2_ NPs, which can be used on large scale for, e.g., TiO_2_ nanotubes [74]. The hydrothermal method is quite expensive due to the increased requirements of high levels of energy, surfactants, pressure, and temperature. Moreover, this a time-consuming process and less yield of TiO_2_ NPs is attained [75]. It has also been reported that changes in the temperature, ranging between 100 and 200 °C, have a lack of control on the size, shape, and crystallinity of the nanotubes. Consequently, the morphology and other properties of the formed NPs are changed, e.g., nanotubes to nanofibers [76]. The chemical synthesis uses high temperature, high pressure, and expensive and toxic chemicals, which produce adverse effects in certain medical applications [77]. 

##### Biological (Bottom-Up) Synthesis 

The bottom-up technique involves biological synthesis via microorganisms (bacteria, algae, fungi, and parasites), green synthesis through plants (roots, leaves, shrubs, herbs, and seeds), and biological synthesis from derivatives (eggshell, albumin, lignin, etc.) [78]. This technique incorporates the atoms or molecules of smaller sizes that undergo reduction or oxidation to generate NPs. These atoms or molecules aggregate biologically, without utilizing toxic chemicals or solvents, to form NPs. These obtained NPs have negligible flaws and defects in their surface morphology, topography, and physico-chemical properties, such as size, shape, crystalline phase form, functional groups, structure, and texture. Above all, TiO_2_ NPs produced by the biological and green routes are immensely biocompatible and biosafe due to the lack of toxic byproducts formed [79]. 

**Figure 4:**
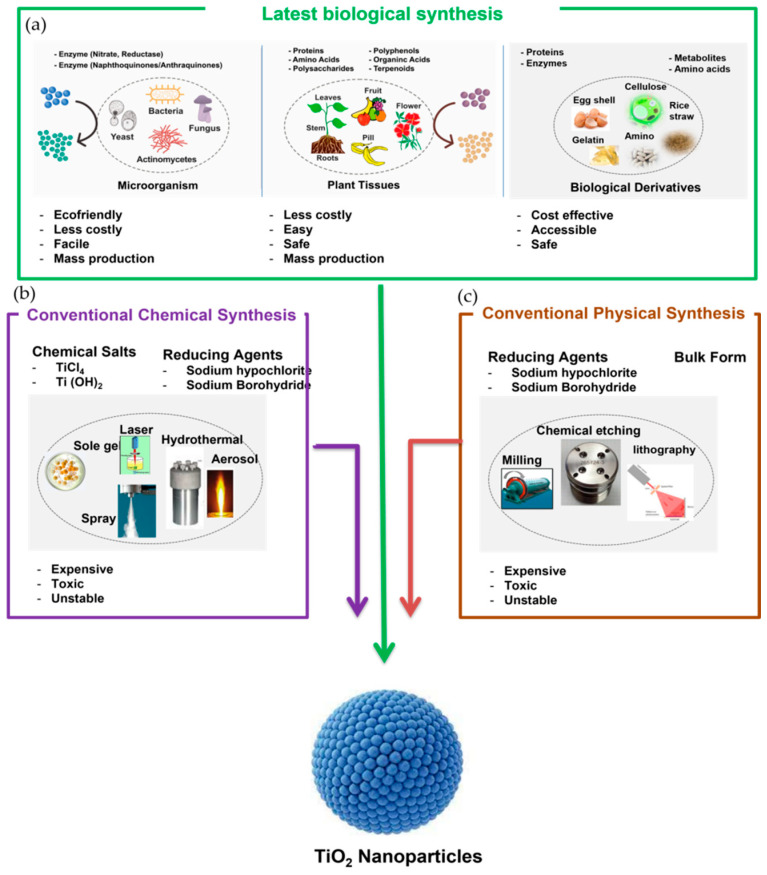
Role of synthesis protocols on advantages and disadvantages of conventional and biological TiO_2_ NPs: (**a**) micro-organisms, plant tissues, and natural derivatives utilized by the latest biological synthesis; (**b**) chemical salts and reducing agents utilized by conventional chemical synthesis; and (**c**) bulk form and reducing agents utilized by conventional physical synthesis (idea adapted from the ref) [86,87].

Similarly, advanced research reflects the successful role of microorganisms in the biological synthesis process because it is easy and less time-consuming. The TiO_2_ NPs formed via different microorganisms are produced in high yields which are very stable, sustainable, cost-effective, biosafe, and biocompatible. This is due to the fact that the capping and reducing agents used are the natural enzymes and proteins present in microorganisms [80,81]. Hence, biological synthesis uses different life forms and organisms from very simple prokaryotic bacterial cells to complex eukaryotic cells such as angiosperms [82]. Different green synthesis methods utilize biological derivatives, such as the polyoxometalates, polysaccharides, Tollens, and phytochemicals (phenols, terpenoids, flavonoids, and cofactors) present in plants, which act as capping and stabilizing mediators in order to improve and enhance the stability of newly formed TiO_2_ NPs [66,83]. The biological derivatives, such as albumin, eggshell, gelatin, lignin cellulose, starch, arginine, peptides, polyamine, lysozyme, pomelo peel, and protamine have been used to synthesize TiO_2_ NPs which are biocompatible, biosafe, and cost-effective (Figure 4).

The direct synthesis method is the most biocompatible technology used for fabricating the TiO_2_ NPs without the consumption of any additional externally introduced templates, capping, reducing agents, etc. Hence, the corresponding natural biological extracts and their derivatives used during the synthesis process can produce a strong capping layer that corresponds to the biocompatibility and stability of these NPs [84]. In addition, this method produces the cost-effective, high-yield mass production of TiO_2_ NPs [67], which are reliable and non-toxic because the method does not utilize high temperature, high pressure, high energy, and toxic chemicals [85].

The biocompatible technology of synthesis is the latest and advanced technique in comparison to the conventional methods. The conventional methods of TiO_2_ NP synthesis are costly and produce adverse effects on human health and the environment [88,89]. The comparative differences between biocompatible and conventional TiO_2_ NPs are given in (Table 1) [85,90,91].

##### Conventional and Biological Synthesis

There are multiple factors that control the synthesis of TiO_2_ NPs and in turn make them suitable for medical dental applications. These significant contributing factors include the total time period, the environmental conditions, the temperature, the synthesis protocols, the solution’s PH, the concentration of extract, and the raw materials used, as is demonstrated.

(i) Solution’s medium pH:

Temperature is also an essential parameter controlling the synthesis of TiO_2_ NPs using three methods. The highest temperature is required by physical methods (>350 °C), while the chemical methodology requires temperature limits below 350 °C. On the other hand, below 100 °C the TiO_2_ NPs are synthesized microbially, and plants use C or ambient temperature. Hence, the nature of the TiO_2_ NPs is determined by the reaction medium’s temperature [92,93].

(ii) Pressure:

The size and shape of biological and green TiO_2_ NPs are greatly influenced by the pressure enforced on the reaction medium. It has been found that the reduction rate of metal ions in biological and green TiO_2_ NPs is faster at ambient pressure due to the utilization of natural biological agents [92,93].

(iii) Time:

The total period for incubating the reaction medium has an immense impact on the type, along with the quality, of the TiO_2_ NPs prepared by biological and green synthesis. Additionally, other factors, including light exposure, synthesis process, and storage conditions, have a great effect on the characteristics of the TiO_2_ NPs synthesized by these routes. The particle aggregation occurs as a result of long-term storage, subsequently resulting in the particle’s growth or shrinkage and enhancing its potency and shelf life [92,93].

(iv) Melting point:

The melting point of TiO_2_ NPs has been controlled by changes in the particle’s shape and size, which eventually influence the properties of these NPs. This means that the melting point of the TiO_2_ NPs has been reduced when the size of these NPs has fallen into the range of the nanometer scale. The shape transformation of those TiO_2_ NPs which display different configurations but have similar energy levels is quite easy. These versatile properties further affect their multiple chemical properties [92,93].

(v) Environment:

The characteristics of TiO_2_ NPs are greatly influenced by the surrounding environment as well. The single TiO_2_ NPs are converted into core-shelled TiO_2_ NPs either through reaction with materials or absorption by materials in the surrounding environment. This process is carried out by corrosion or oxidation occurring in the environment. The biological TiO_2_ NPs are thicker and larger in size due to the formation of the coating around them [38] that might be responsible for their potent characteristics. In a study, zinc sulfide NPs changed their crystalline nature instantly with a change in environment from wet to dry. In another study, cerium-nitrate NPs changed their chemical properties when peroxide was used in their suspension medium [92,93].

### 3.2. Characterization Techniques of TiO_2_ NPs

**Figure 5:**
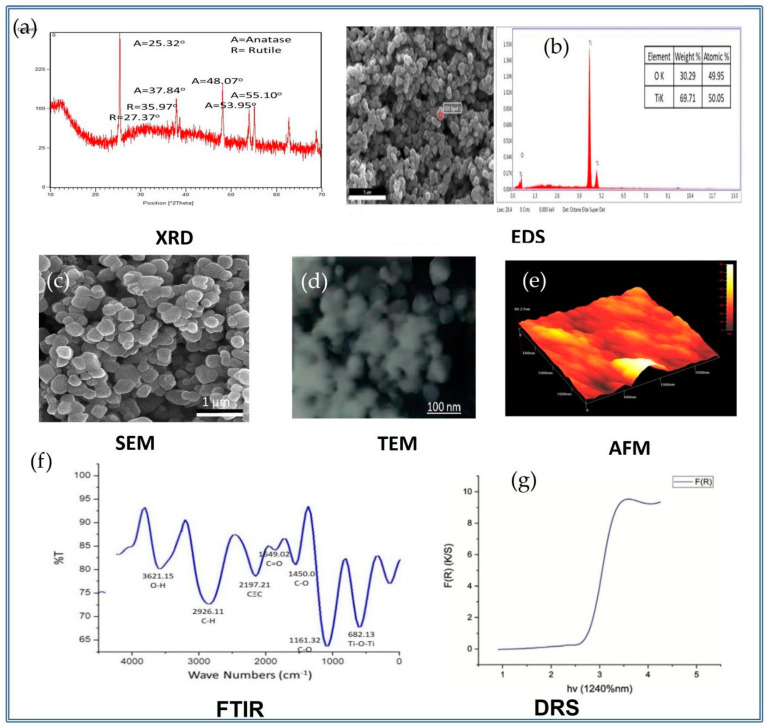
Different characterization techniques utilized by TiO_2_ NPs for their particular features: (**a**) X-ray diffraction pattern spectroscopy (XRD) for investigating the size, shape, and phase form; (**b**) energy-dispersive X-ray spectroscopy (EDS) for investigating the elemental composition; (**c**) scanning electron microscope (SEM) for investigating the size, shape, and surface morphology; (**d**) transmission electron microscope (TEM) for investigating the size and shape; (**e**) atomic force microscope (AFM) for investigating the surface roughness and topography; (**f**) Fourier transform infrared spectroscope (FTIR) for investigating the functional groups; and (**g**) diffuse reflectance spectroscope (DRS) for investigating the size, shape, and crystal type [79].

The TiO_2_ NPs are characterized by their size, shape, phase form, crystallinity, surface roughness, morphology, texture, topography, elemental composition, band-gap absorbance energy, and compounds and the functional groups present in them that are the key features responsible for their biocompatibility. These standardized tools render them biocompatible and enhance their potency for utilization in medical and dental applications (Figure 5) [79,94,95,96]. 

#### 3.2.1. Scanning Electron Microscopy (SEM) and Transmission Electron Microscopy (TEM) for Size and Shape

The size, shape, structure, and surface morphology of the TiO_2_ NPs are determined by scanning electron microscopy (SEM), which is a basic parameter for characterizing NPs. SEM is a powerful technique for obtaining magnified high-quality and high-resolution images with a good depth of field to provide information about the morphology of the TiO_2_ NPs [97,98]. The standardized morphological analysis tool, named the transmission electron microscope (TEM), gives a more refined knowledge regarding the crystallographic arrangement of the atoms, complex phase transitions, elemental composition, dislocations, selected area electron diffraction (SAED), and defects in the TiO_2_ NPs [97,98].

**Figure 6:**
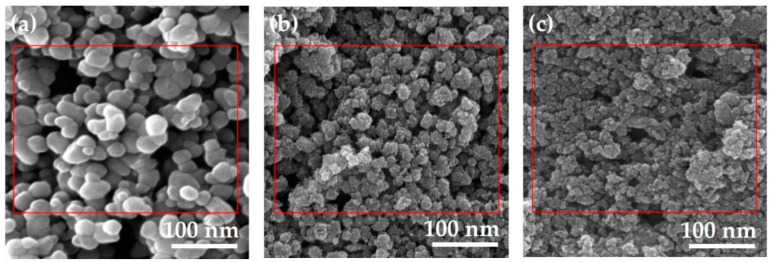
SEM images of different sizes of TiO_2_ NPs attained from (**a**) *Bacillus subtilis* [79], (**b**) *Cassia fistula*, and (**c**) hydrothermal heating [80].

**Figure 7:**
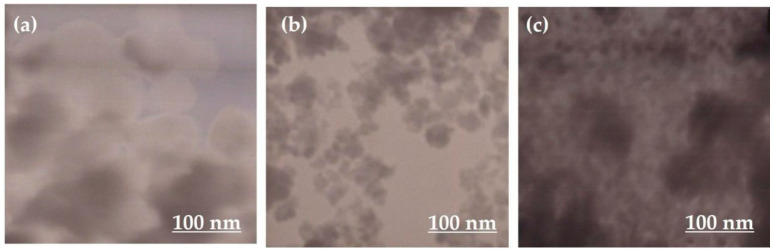
TEM images of different sizes of TiO_2_ NPs attained from (**a**) *Bacillus subtilis* [79], (**b**) *Cassia fistula*, and (**c**) hydrothermal heating [80].

The studies conducted by A. Mansoor et al. revealed the different sizes and shapes of TiO_2_ NPs derived from *Bacillus subtilis*, *Cassia fistula*, and hydrothermal heating. These different sizes and shapes of these NPs had a great influence in developing the cytotoxicity. The TiO_2_ NPs prepared from *Bacillus subtilis* were purely natural, having a slightly large size and spherical shape that prohibited their gross adsorption and absorption in the cell surfaces coming into contact with them. Thus, these TiO_2_ NPs were biosafe and biocompatible for their medical and dental applications (Figure 6a and Figure 7a). On the other hand, the TiO_2_ NPs formed by *Cassia fistula* and hydrothermal heating were very much smaller in size, and the irregular shapes were easily absorbed and adsorbed in the cell surfaces, resulting in cytotoxic reactions. Therefore, these newly formed NPs could not be used in nanomedicine and nanodentistry (Figure 6b,c and Figure 7b,c) [79,99]. These NPs could be recommended for medical and dental applications only if changes were made in the time, pH, melting point, chemicals, substrates, high temperature, and pressure during the synthesis protocols in future.

#### 3.2.2. X-ray Diffraction Powder Spectroscopy (XRD) for Crystalline Phases

**Figure 8:**
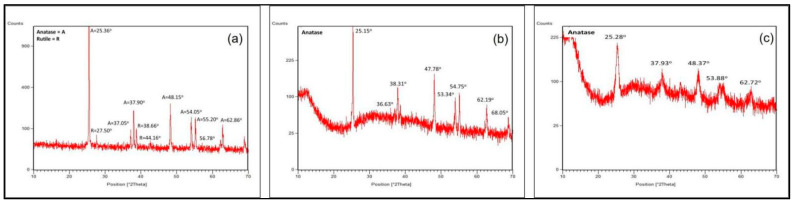
XRD images of different sizes of TiO_2_ NPs attained from (**a**) *Bacillus subtilis* [79], (**b**) *Cassia fistula*, and (**c**) hydrothermal heating [80].

The X-ray diffraction pattern (XRD) is utilized to investigate the crystallography and structural arrangement of TiO_2_ NPs [100]. XRD is a fast and indispensable analytical technique, which is mainly used for a material’s identification, characterization, crystal content, phase identification, quality control, lattice plane spacing, purity percentage, and epitaxial growth of crystallites [101,102]. The XRD methods utilizing powder can easily identify the crystalline, crystallite, semi-crystalline, and amorphous forms of materials [103]. Recently, the role of the crystalline phases of TiO_2_ NPs in the medical and dental applications were investigated by A.Mansoor et al. in 2022, where it was revealed that the mixture of the anatase–rutile phase form state attained by the TiO_2_ NPs formed by the *Bacillus subtilis* (Figure 8a) were the best for certain medical and dental applications as compared to those prepared by *Cassia fistula* and hydrothermal heating, where only the anatase phase form state was dominant (Figure 8b,c). This happened because of the comparatively high temperatures and pressures used in the synthesis of both of the latter TiO_2_ NPs. As a matter of fact, the anatase phase form state is highly thermally reactive and unstable while the rutile phase form state is the most thermally stable and is less reactive [79,99]. Efforts should be made to produce the rutile phase form state of the TiO_2_ NPs in order to induce them in the future nanomedical and nanodental procedures.

#### 3.2.3. UV–Vis Diffuse Reflectance Spectroscopy

**Figure 9:**
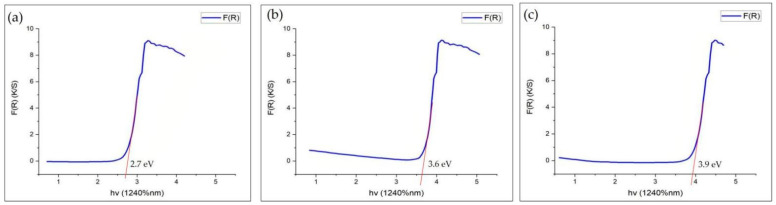
DRS images of different sizes of TiO_2_ NPs attained from (**a**) *Bacillus subtilis* [79], (**b**) *Cassia fistula*, and (**c**) hydrothermal heating [80].

The ultraviolet (UV) visible spectrum records the reflectance and absorbance in the 300 nm to 800 nm wavelength range in order to investigate the alterations in the molecular energy levels of TiO_2_ NPs. Electron transfer from either π-bonded orbitals or non-bonded orbitals originates this form of alterations. Thus, this standard tool [104] obtains deep knowledge about aromatic and non-aromatic compounds, π-electron orbital systems, conjugated and non-conjugated peripheries, bonding and non-bonding attachments, and saturated and unsaturated electronic systems in TiO_2_ NPs. Moreover, the UV–Vis absorption spectrum depicted the two characteristic peaks of TiO_2_ NPs in the wavelength and absorbance ranges of 264 nm and 322 nm in previous studies [105,106]. The aqueous solutions used in UV–Vis spectroscopy are responsible for confirming the formation of TiO_2_ NPs [106,107]. The standard of the band gap energy for the NPs is 3.20 e.V, which means that the size of the NPs is somewhat big if the calculated value is less than the standard, but if the calculated value is greater than the standard, the size of the NPs is very small. Various researchers concluded that TiO_2_ NPs generated by *Bacillus subtilis* showed 2.7 e.V, confirming the somewhat big and safe size of TiO_2_ NPs for their employment in the fields of medicine and dentistry (Figure 9a). Additionally, the TiO_2_ NPs fabricated by *Cassia fistula* and hydrothermal heating depicted 3.6 e.V and 3.9 e.V values, which showed the extremely small size of these NPs (Figure 9a,b) [79,99]. It might be possible to control the large band gap energy values as compared to the standard after the advancements in the conventional synthesis processes.

#### 3.2.4. Fourier Transform Infrared Spectroscopy (FTIR)

**Figure 10:**
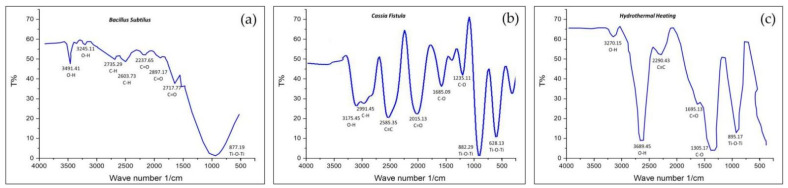
FTIR images of different sizes of TiO_2_ NPs attained from (**a**) *Bacillus subtilis* [79], (**b**) *Cassia fistula*, and (**c**) hydrothermal heating [80].

FTIR is the standard characterization tool that is helpful in getting the infrared spectra of the absorption, adsorption, emission, and photoconductivity of semi-solids, gases, solids, and liquids by determining the nature of their bonds and functional groups [97]. The functional group available in a TiO_2_ nanoparticle is recorded between 4000 and 400 cm^−1^ in FTIR spectroscopy [86,108]. The stretching and bending peaks of the Ti–O–Ti vibration which appear in the range of 1000–1300 cm^−1^ mainly correspond to the formation of TiO_2_ NPs in addition to other organic compounds, including nitro compounds, ethers, esters, H-O-H bending, polyphenols, hydroxyl (OH) groups, and amide-II [109,110]. The current research concludes that the TiO_2_ NPs formed by *Bacillus subtilis* reveal only a few functional groups, such as O-H, C-O, and C-H (Figure 10a), whereas several functional groups such as O-H, C-O, C-H, C=O, C-C, C=C, and C≡C were observed in the TiO_2_ NPs prepared by *Cassia fistula* and hydrothermal heating (Figure 10b,c). These functional groups are responsible for the development of the cytotoxicity of these NPs. Thus, the presence of a few functional groups in the TiO_2_ NPs formed by *Bacillus subtilis* makes them the best option to be utilized for the medical and dental fields as a result of their non-cytotoxicity [79,99]. The TiO_2_ NPs generated by *Cassia fistula* and hydrothermal heating could be modified by using the least reactive chemicals in order to reduce the overproduction of large amounts of functional groups that could be harmful in medicine and dentistry. 

#### 3.2.5. Elemental Composition Analysis (EDX)

**Figure 11:**
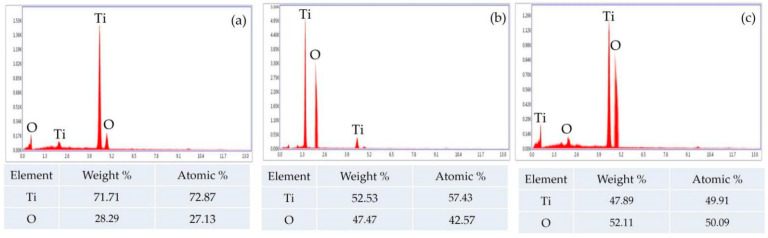
EDX images of different sizes of TiO_2_ NPs attained from (**a**) *Bacillus subtilis* [79], (**b**) *Cassia fistula*, and (**c**) hydrothermal heating [80].

The energy dispersive X-ray spectrometer (EDX or EDS) is a versatile piece of equipment used to identify the spectrum and determine the elemental composition present in TiO_2_ NPs [111]. This elemental composition gives complete information regarding the purity and performance of these TiO_2_ NPs [112]. In recent studies, it was observed that TiO_2_ NPs prepared by *Bacillus subtilis* revealed a high content of titanium but a low content of oxygen (Figure 11a). On the other hand, TiO_2_ NPs formed by *Cassia fistula* and hydrothermal heating depicted a large content of oxygen but a lesser content of titanium (Figure 11b,c). The presence of large amount of available titanium in the TiO_2_ NPs formed by *Bacillus subtilis* might have enabled them to be utilized easily in the medical and dental areas as compared to the TiO_2_ NPs formed by *Cassia fistula* and hydrothermal heating, where the content of titanium was too low. This imbalance between the weight% and atomic% of titanium and the oxygen in the TiO_2_ NPs formed by *Cassia fistula* and hydrothermal heating might be the reason for their non-consumption in medicine and dentistry [79,99]. The volume and concentration (dose) of the chemicals must be controlled efficiently to produce TiO_2_ NPs with nearly equal or a somewhat large weight% and atomic% of titanium in comparison to the oxygen.

#### 3.2.6. Atomic Force Microscopy Analysis (AFM)

**Figure 12:**
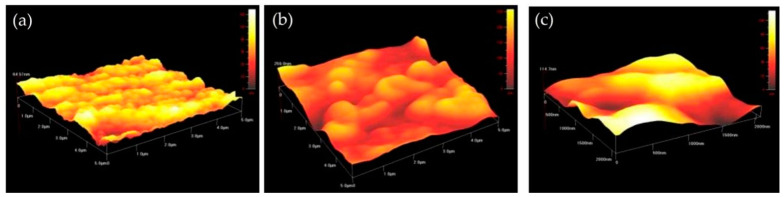
AFM images of different sizes of TiO_2_ NPs attained from (**a**) *Bacillus subtilis* [79], (**b**) *Cassia fistula*, and (**c**) hydrothermal heating [80].

The most useful and popular standard tool employed to image the surface topography, texture, and structure of TiO_2_ NPs is the atomic force microscope [113]. It gives entire, detailed information about the topographic map, including the surface roughness, bumps, ridges, and phases of these NPs [114,115]. According to the current research, the TiO_2_ NPs prepared by *Bacillus subtilis* displayed bumps of the anatase–rutile phase form states with minimum roughness (Figure 12a), as compared to the TiO_2_ NPs formed by *Cassia fistula* and hydrothermal heating (Figure 12b,c) where they showed enhanced roughness with slight or no bumps in the anatase phase form state. This minimal roughness might have allowed the least absorption and adsorption of these NPs in the cell surfaces, thus making them biocompatible for medical and dental applications. The maximum surface roughness in the TiO_2_ NPs formed by *Cassia fistula* and hydrothermal heating might have allowed the increased level of absorption and adsorption of these NPs in the cell surfaces, resulting in their cytotoxicity [79,99]. The large amount of surface roughness present in the TiO_2_ NPs formed by *Cassia fistula* and hydrothermal heating could be controlled by utilizing the large amounts of stabilizing agents. These agents could be either natural or synthetic and form a capping layer on the surface of these NPs, making them suitable for applications in the medicine and dentistry.

#### 3.2.7. Characteristic Features of TiO_2_ NPs Dependent on Characterization Techniques

**Figure 13:**
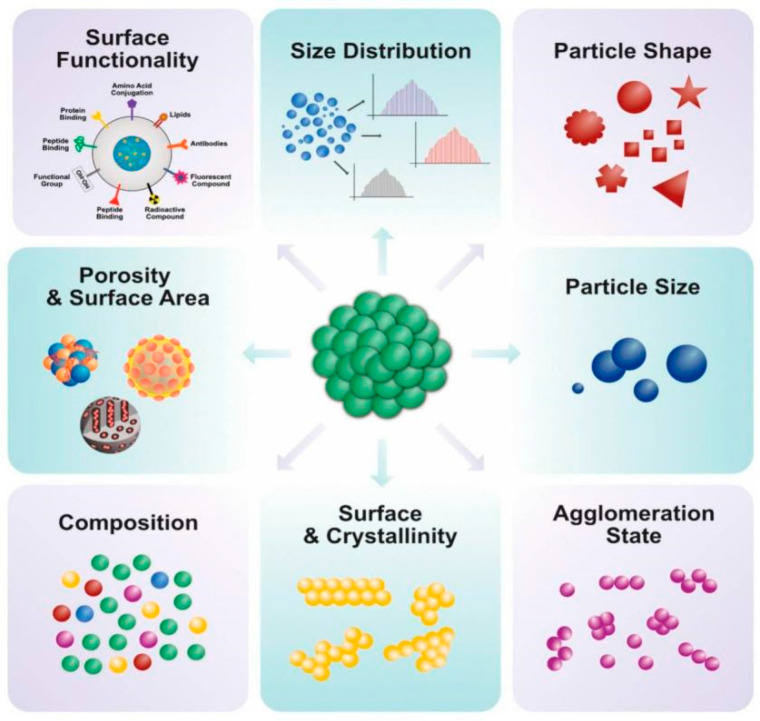
Characteristic features of TiO_2_ NPs dependent on characterization, showing the surface functionality, size distribution, particle shape, porosity, surface area, particle size, composition, surface, crystallinity, and agglomeration state [119].

The unique physical, chemical, mechanical, and biological properties of TiO_2_ NPs make them superior to their parent bulk material from which they are formed. These ideal properties make them compatible and increase their demand in health care systems [116]. There is strong correspondence between these various properties of NPs and their utilization in the fields of medicine and dentistry. Other important parameters include dimension, durability, quality, number, quantity, mass, bulk, volume, and aggregation (Figure 13) [117,118].

##### Role of Size and Shape of TiO_2_ NPs on Their Medical and Dental Applications

The nanometer size of TiO_2_ NPs provides them with a high surface energy, a large fraction of surface atoms, reduced imperfections, spatial confinement, and reduced imperfections in comparison to the corresponding bulk material. At the nanometer scale, the properties of NPs become size- and shape-dependent, which involves chemical properties (reactivity), catalysis, thermal properties (melting temperature), mechanical properties (adhesion and capillary forces), optical properties (absorption and scattering of light), electrical properties (current), and magnetic properties (superparamagnetic effect) [120,121,122,123,124,125,126]. Early in 1996, Volokitin et al. [127] already confirmed that quantum size strongly influences the thermodynamic properties of metallic origin-based NPs especially TiO_2_ NPs. The confinement of movement of the electrons leads to the formation of discrete energy levels and electric dipoles because of the smaller size of newly formed NPs. This small size of NPs greatly influences their oxidation, reduction, and catalytic properties in a positive manner, thus reducing cytotoxicity [128]. Thus, because of these extraordinary features the TiO_2_ NPs are of great interest to the current researchers [129].

The size has been considered as one of the most important parameters responsible for inducing the toxic effects on human health. The much smaller-sized TiO_2_ NPs have a higher surface-area-to-volume ratio, with higher chemical reactivity and vice versa. Due to the large surface area of these very small-sized TiO_2_ NPs, more of them are absorbed in its surface, resulting in toxicity [130]. In an in vivo study, rats consumed two sizes of TiO_2_ NPs, with diameters of 20.27 nm and 250.10 nm; the study concluded that smaller TiO_2_ NPs have bit more of an adverse cytotoxic effect on lungs in comparison to large-sized TiO_2_ NPs of 250.10 nm [131]. A study conducted previously revealed a strong direct correlation between TiO_2_ NPs size and level of DNA damage [117,118]. The smaller TiO_2_ NPs are more easily taken up by cells, producing intense adverse effects on cell function, thereby leading to a high level of toxicity. The reactive oxygen species (ROS) production is related to nano-toxicity [132].

Moreover, particle shape also plays a dominant role in determining the toxicity of TiO_2_ NPs [133]. Different shapes of TiO_2_ NPs display different cellular uptakes, subcellular localization, and production of reactive oxygen species [134]. There exists a direct correlation between the shape of the TiO_2_ NPs and cytotoxicity. There are only limited studies available that have investigated biocompatibility in cell lines. Braydich-Stolle et al. [135] observed the effects of size and crystal structure on toxicity or mode of cell death. In another study, Gratton et al. investigated the influence of shape on toxicity and revealed that non-spherical TiO_2_ NPs (rod-shaped particles) are easier and faster to become absorbed/adsorbed on cell surfaces, thus making them cytotoxic in comparison with other TiO_2_ NP shapes (spherical-shaped particles) [136].

##### Role of Surface Area of TiO_2_ NPs on Their Medical and Dental Applications

The ratio of the surface atomic number to the total number of atoms increases greatly as the particle size gets smaller in NPs [121]. This decrease in particle size increases the number of atoms on the surfaces as compared to those found inside, which leads to the enhanced chemical reactivity of these NPs [137]. As a result, the surface-to-volume ratio of the NPs also increases, which has a significant effect on their physical, chemical, and mechanical properties compared to those of the corresponding bulk state in the parent material [122,123,124,125,126,137,138]. In addition, TiO_2_ NPs show increased catalytic activity as compared to other NPs [124,126]. The reason it became possible was because the smaller size of TiO_2_ NPs provides a large surface area for the catalysis to take place [126,139,140,141,142]. If the TiO_2_ NPs get very much smaller, then they exhibit a much greater surface area and number of particles per unit mass, which leads to much greater chemical reactivity than is even required [143]. This greater activity eventually increases the absorption of these NPs in the cell lines, elevating reactive oxygen species (ROS) production [133], oxidation [144], and DNA damage [133].

##### Role of Crystalline Phases of TiO_2_ NPs on Their Medical and Dental Applications

Owing to the many active atoms on the surface of NPs, these NPs have specific enhanced surface adsorption, dispersion, aggregation, viscosity of colloidal suspension, and rheological properties [145]. Chemically, these NPs are more active and stable than the corresponding bulk material used for their formation [146]. The three important phase states of the TiO_2_ NPs are brookite, anatase, and rutile [122,123]. These crystal phases are available in the form of octahedra with six oxygen anions surrounded by titanium cations. The formula of these NPs is TiO6/3, which is equal to TiO_2_ leading to different patterns of crystalline phase states [123]. These three distant phase form states differ in structure, texture, size, density, volume, and refractive index [122,123,124]. The rutile phase form state is well known for its high level of texture, structure, and chemical stability during the preparation methods, but its use is rare [122,123,124,125,126,147]. The anatase and brookite phase form states are meta-stable because they are thermodynamically unstable as compared to the rutile phase form state when used in the industry [125,126]. The brookite is rarely used in the industry because it is difficult to prepare [123]. The chemical reactivity of anatase is the highest when compared to the other phases [148,149]. In a study, Sayes et al. [149] reported that TiO_2_ NPs (80/20; anatase/rutile, 3–5 nm; 100 μg/mL) produced 6 times higher reactive oxygen species (ROS) than rutile after UV exposure. This is because the anatase phase state of TiO_2_ NPs produces ROS when exposed to ultraviolet light [149]. It is even suggested that TiO_2_ anatase has a greater toxicity potential than TiO_2_ rutile [150,151]. In addition, Di-Virgilio et al. evaluated the impacts of the anatase phase form states in different dose volumes, e.g., (0–300 μg/mL) on rat osteosarcoma-derived cell lines. Eventually, they reported the formation of phagocyte vesicle-induced cytotoxicity leading to osseointegration failure [152]. In another study, Martinez-Gutierrez et al. reported the effect of combining (anatase and rutile) TiO_2_ phase form states with silver NPs. They observed that the combination of two phases showed a superior antimicrobial activity with reduced cytotoxicity as compared to 100% rutile TiO_2_ NPs due to their inherent properties [153]. A recent study found that TiO_2_ NPs with a large percentage of anatase (anaerobic P-25 99.5% 73–85% anatase, 14–17% rutile, and 2–13% amorphous) produced genotoxicity, mutagenicity, and cytotoxicity on human fibroblasts in different doses (such as 10, 25, 50, 100, 250, 500, and 1000 μg/mL) to detect γ-H2AX expression [154].

##### Role of Dose of TiO_2_ NPs on Their Medical and Dental Applications

The TiO_2_ NPs can go directly into the liquid phase of the reaction system without getting attached to an inert carrier that could have adversely affected their catalytic activity, stability, and reactivity [155]. This is because metal oxide NPs, especially TiO_2_ NPs, show a much higher catalytic activity than any other type of NPs due to the fact that the small nano-size and the large surface area of these NPs support an exceptionally increased active catalytic activity [156,157]. The dose is directly related to the degree of exposure or concentration of the substance in the medium (such as air, food, and water). The different dosage indicators have been used in nanotoxicology, including cm^2^/mL, μg/mL, μg/cm^2^, and the number of particles/mL [158]. Previously, a study revealed that the cytotoxic effects of TiO_2_ NPs after inhalation were more when exposed to small-sized TiO_2_ NPs (20 nm diameter) with a low dose (10 mg/m^3^) as compared to large-sized TiO_2_ NPs (300 nm diameter) with a high dose (250 mg/m^3^) [130]. In another study, cell viability was reduced by 20–30% when HT29 cells were exposed to different concentrations of TiO_2_ (50, 100, 200, and 400 µg/mL) for 48 h; the study concluded that these nanoparticles affect cell viability, resulting in cytotoxicity in a dose-dependent manner [159]. 

##### Role of Surface Coating of TiO_2_ NPs on Their Medical and Dental Applications

The metallic NPs possess unexceptional optical properties as a result of their nano-size ranging between 1 and 100 nm and the confinement of their electrons together with the quantum effects, e.g., in solution form gold (Au) NPs are found as deep red and/or black [160]. The interaction of light and the coupling between several metallic NPs, especially TiO_2_ NPs, induce a field enhancement in the surroundings, resulting in increased absorption, scattering, and luminescence, thus giving them whiteness [161]. The TiO_2_ NPs are considered as the cosmetically most white pigments, which can easily enhance the aesthetics in medical and dental applications [162,163]. The (TiO_2_) NPs are white in color, with the highest refractive index of 2.50 in the anatase phase form state and 2.70 in the rutile phase form state, which is the whitest in color [122,147]. The TiO_2_ NPs are capable of undergoing photocatalysis because of their dual behavior of absorbing and scattering ultraviolet radiation against light [122,123,124,164]. 

In the cases where TiO_2_ NPs without surface coatings are used, they initiate the cytotoxic reactions when they come into contact with cells and other biological materials. The development of surface coatings on these NPs can make them non-toxic via the utilization of surfactants. Eventually, the physical and chemical properties of the TiO_2_ NPs are changed, which prohibits the cytotoxicity. The important properties in this respect are the magnetic, electrical, thermal, and optical properties and the chemical reactivity [165,166]. Moreover, the presence of oxygen, (ozone) [166], oxygen free radicals [167], and transition metals [144] on the surface of the initiating TiO_2_ NPs can lead to the formation of reactive oxygen species (ROS), which causes inflammation. Previously, a study was carried out which concluded that cytotoxic reactions in silica are due to reactive oxygen species and surface free radicals [130].

The metallic NPs have higher values of hardness, plasticity, specific heat, thermal expansion, conductivity, diffusivity, and lower values of sintering temperature and shrinkage as compared to the bulk material [155,168]. Other properties of metallic NPs, especially TiO_2_, include surface plasmon resonance and superparamagnetism, which makes them desirable as diagnostic intervention tools for medical applications [169,170,171].

### 3.3. Significance of TiO_2_ NPs in Medical and Dental Platform

Previously, the utilization of other NPs such as graphene was wide because of its versatile characteristics, including outstanding biocompatibility, physicochemical properties, and antimicrobial activity. Furthermore, it was highly conductive with a large surface area; it was wear/friction resistant and could stabilize the NPs if used as a substrate [172]. Additionally, graphene was a material of choice for various dental applications such as in bone grafting, tissue engineering, pulp and periodontium regeneration, dental cements and adhesives, resins, whitening agents, dental implants collagen membranes, and biomarkers of oral saliva. The use of graphene in dentistry resulted in enhanced positive outcomes, but its manufacturing for industrial purposes was quite challenging and time-consuming [173]. This was an initial step that opened a gateway to the incorporation of other biocompatible and cost-effective nanomaterials in the future nanodentistry. Among them, a TiO_2_ nanoparticle was one of the top five most common, easily available, and compatible NPs for the industry purposes [140,174,175]. There has been a persistent rise in the production of TiO_2_ NPs globally, which increases its mass production availability for different fields [176]. Currently, TiO_2_ NPs are the most biocompatible, with low toxicity and low allergic potential that makes them ideally suitable for utilization in medical and dental applications [39,40,177,178]. The TiO_2_ NPs are resourceful, versatile, and adaptable because of their extraordinary functionality, increased strength, light weight [179], brightness, resistance to UV light, high density [177,178,180], cost effectiveness, purely white color, high corrosion resistance [181,182,183], long-term chemical stability, and durability [184,185]. In addition, these NPs possess high mechanical resistance, high electrical conductivity, low thermal diffusivity, chemical stability, corrosion and wear resistance, and low thermal conductivity. These NPs are ductile in pure form and can be easily shaped into any form and type that makes them fatigue resistant [115].

The desired mechanical and physicochemical properties of TiO_2_ NPs in the context of size, shape, surface texture, morphology, topography, crystalline, and phase form can be easily attained which makes them superior when compared to other metal oxide NPs [42]. According to the previous literature, the low ion release and stability of these TiO_2_ NPs additionally accounts for their biocompatibility. These NPs are capable of producing a TiO_2_ layer on their surface after their accidental exposure to air, thus preventing the metal inside these NPs from being dissolved and getting distorted. This chemically inert layer of TiO_2_ plays a key role in the biosafety and biocompatibility of these NPs in the human body [181,186,187]. Owing to these versatile features, the use of TiO_2_ NPs in the dental and medical field has rapidly started to increase [182,188,189]. According to previous research, large quantities of pure titanium in the range of about 99.5% and 0.5% of the interstitial elements (carbon, oxygen, and nitrogen,) are even safe, biocompatible, and allergic free in both dental and medical applications. This research concluded that even significantly more large quantities of titanium oxide are required for any allergic or toxic reaction to arise in humans [182]. 

#### 3.3.1. Significance of TiO_2_ NPs as a Medical Material

**Figure 14:**
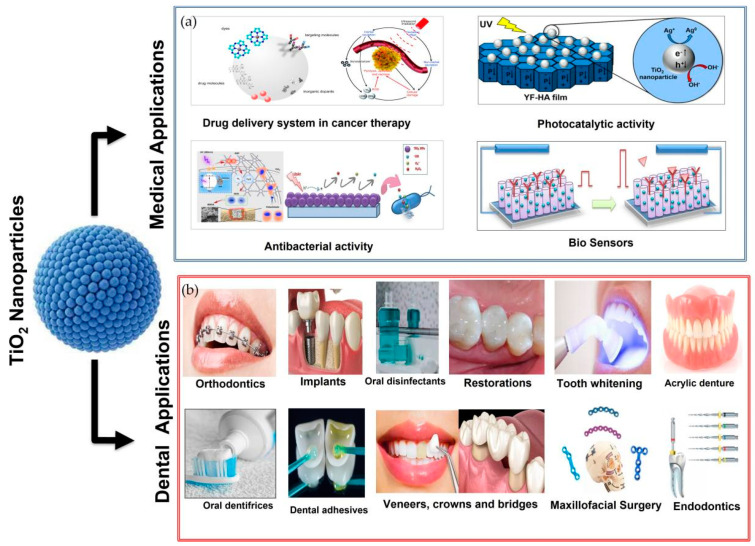
Medical and dental applications of TiO_2_ NPs: (**a**) drug delivery in cancer therapy, photocatalytic activity, antibacterial activity, and bio sensors in medical field; (**b**) orthodontics, implants, oral disinfectants, restorations, tooth whitening, acrylic dentures, oral dentifrices, dental adhesives, veneers, crowns, bridges, maxillofacial surgery, and endodontics in dental field (idea adapted from the ref [351,352]).

The medical applications of the TiO_2_ NPs is illustrated in the Figure 14a.

##### Use of TiO_2_-Based Drug Delivery Systems in Cancer Therapy

The (TiO_2_) NPs have potential biomedical and industrial applications due to their size, acceptable biocompatibility, lower toxicity, and cost effectiveness [190,191,192]. Nanostructured arrays comprising nanotube walls of TiO_2_ involving copper and silver oxide NPs have been used as surface coatings due to their antibacterial nature. Moreover, they have favorable cytocompatibility and are utilized for differentiation, cell spreading, and proliferation [193,194]. Traditional chemotherapeutic drugs have severe adverse effects on normal cells, due to which nanotechnology has gained significant importance in the diagnosis and treatment of cancer [195,196]. 

The role of nanotechnology in cancer treatment is related to the enhancement of therapeutic effects and the reduction in the side effects of chemotherapeutic drugs [197]. Moreover, controlled drug delivery and targeted drug release are aimed at through the use of these NPs. Previous studies investigated the TiO_2_ whiskers in cells for the delivery of drugs in cancer therapy and the synergistic effects on accumulation and internalization of daunorubicin [198]. Similarly, a synergistic response has been observed in cell lines involving breast cancer when the (TiO_2_) NPs conjugate to doxorubicin [199]. 

A study conducted on TiO_2_ nanotube surfaces containing quantum dots of zinc sulphide, which were studied with 5-flourouracil, revealed that it was of significant importance as a drug delivery system [200]. The studies revealed that NPs with a modified surface may enhance the performance of anticancer medications and lower their toxicity [195]. The biochemical and hematological results of a study revealed better anticancer performance of surface-altered paclitaxel affixed to TiO_2_ NPs and modified hydroxyapatite as compared to pure paclitaxel [201]. Similarly, a higher accumulation of cisplatin was observed in cancer cells when cisplatin was loaded on TiO_2_ NPs containing hyaluronic acid [201]. 

The TiO_2_ NPs may also serve as a photoactive carrier for drug delivery. These NPs may be a suitable carrier for various drugs due to their stability, high surface area, possibility for surface modification, and availability [202]. Previously, studies were carried out for developing skin cancer treatment and light-controlled drug release by the modification of TiO_2_ NPs with the help of polyethyleneimine (PEI), which may act as an agent for targeting cancer [203]. In another study, drug delivery triggered by near-infrared radiation was developed through the loading of DOX for the preparing of a porous TiO_2_ shell. Furthermore, hyaluronic acid capping was performed, and the release of the drugs was studied. This study also declared that at lower doses of the drug a decline in cell viability meant that it was a promising method for therapy in cancer [204]. 

Colloidal TiO_2_ NPs that are controlled through light may be used for delivering ruthenium complex to melanoma cancer cell lines. This system demonstrated a fast release profile when an ultraviolet light was used in comparison to red light. It has been pointed out that the destruction of cancer cells may be higher due to elevated oxidative stress when exogenous agents are used, as the reactive oxygen species are higher in the tumor environment [205]. Apoptosis is triggered in tumor environments when reactive oxygen species are induced, and it is an appropriate cell death type in cancers [206]. When stimulated with ultrasonics, TiO_2_ NPs exhibit improved anticancer activity against various anticancer cell lines. [205,207] In sonodynamic therapy, hydrophilized TiO_2_ NPs demonstrate higher resistance to degradation and prolonged circulating time [207]. Radiofrequency as a no noninvasive technology may help in the release of drugs from nanotechnology-based TiO_2_ drug-delivery systems. Drug release can be induced in titania NPs (TiO_2_) by transferring radiofrequency energy through efficient thermal transducers such as gold NPs [208]. This technique has displayed substantial capability in inhibiting human gastrointestinal and hepatocellular cancer cells [209].

##### Prevention and Treatment of Infections through TiO_2_ Based Antibacterial Devices

With the advancements in nanotechnology, metals and metallic oxides have gained importance in biomedical industry as they can be used as effective antimicrobial agents due to their enhanced safety and stability as compared to organically produced antimicrobial agents [210,211]. Initially, TiO_2_ was used as a photocatalytic microbicide against *Lactobacillus acidophillus*, *E*. *coli*, and *Sacharromyces cerevisiae* [212]. Several studies suggest that in water the photocatalytic activity of TiO_2_ is effective against various organisms, such as bacteria (Gram-positive and Gram-negative), viruses, algae, fungi, bacterial toxins, and protozoa [213]. In water and in response to exposure from UV light the microbial cell viability may be inhibited by a thin film of TiO_2_ and platinum as proven in previous studies [212].

##### Antibacterial Action of TiO_2_ in Orthopedic Implants

Osteomyelitis may be caused due to an infection with mostly bacteria or due to fungi. This infection typically occurs as a consequence of the treatment with implants required in the case of bone surgery or bone fracture. The management of this condition requires a specific and prolonged treatment with antibiotics. This infection may be prevented by the use of implants with modified surfaces such as implants coated with TiO_2_ nanostructures [214]. One study revealed the antibacterial properties demonstrated by titanium-based implants when silver-doped TiO_2_ nanotubes were used for the surface modification of these implants [215]. This study explained that the adhesion of bacteria reduced when the surface of the titanium-based implants was loaded with TiO_2_ as compared to non-coated implants.

Recently, micro-arc oxidation composites with incorporated (TiO_2_) were coated on magnesium alloy, and this was presented as a biomaterial with antibacterial properties against microorganisms such as *Escherichia coli*; it demonstrated sufficient bioactivity with in vivo degradation properties and acceptable cytocompatibility for applications in orthopedic surgeries [216]. Metal wires and pins are used in cases of orthopedic fractures for the purpose of fixation. These devices are susceptible to colonization with bacteria such as *E*. *coli*, *Staphylococcus aureus*, and *Staphylococcus epidermidis* [217]. The TiO_2_ coating of these pins and wires is effective due to its photo killing activity as it influences bacteria in comparison to the host tissue. Moreover, TiO_2_-coated implants and devices are biocompatible and environmentally safe [218]. Moreover, osteogenic differentiation is promoted by TiO_2_ NPs [219]. A study conducted on implants produced two implant surfaces (Ti-160 and Ti-120) using the electrophoretic deposition method. These treated surfaces enhanced osteoblast proliferation and reduced bacterial colonization [220].

##### Antibacterial Applications of TiO_2_ in Hospitals and Medical Devices

In medical devices, the formation of microbial biofilms poses a challenge for the health professionals. These biofilms are mostly formed by Staphylococcus aureus and Staphylococcus epidermidis and may be the source of infection. The hospital industry may employ TiO_2_ nanoparticle-coated surfaces due to their antibacterial properties and eliminate these sources of infection as traditional methods of disinfection are not as beneficial when compared to the photocatalytic methods [221]. The application of TiO_2_ NPs has been performed on ceramic tile surfaces and Petri dishes, and it has been reported that the deactivation of various microorganisms can occur in response to ultraviolet light [222]. Light-induced disinfection can be performed in TiO_2_-coated catheters, and these are therefore safe to use as biomedical equipment. A study conducted on TiO_2_-coated catheters revealed the reduction in Escherichia coli after the exposure to UV light within 1 h [223]. Bactericidal effects shown by medical tubes and silicone catheters on cells of Escherichia coli in a study were observed [224]. 

Self-monitoring devices for blood glucose in diabetes (SMBG) contain sterile needles known as lancets. The ability to self-sterilize is an issue with these needles. These needles have previously been sterilized with gamma ray radiation that poses a drawback of gamma ray leakage and higher cost of equipment. Therefore, the photocatalytic activity of TiO_2_ NPs that provides antibacterial properties to the coated surfaces has been used to resolve this issue. Under ultraviolet illumination, TiO_2_ coated lancets demonstrated antibacterial properties to the K-12 suspension of Escherichia coli [225]. Sulphur-doped TiO_2_ has also been used in medical equipment for the purpose of antimicrobial activity. In order to control hemorrhage and infection it has been proposed that metal oxides such as TiO_2_ may be incorporated into polymers. Hemostatic ability and antibacterial activity have been enhanced previously by the use of nanocomposite films comprising chitosan pectin containing TiO_2_ NPs. These could serve as basis for the production of wound healing bandages.

##### TiO_2_ Implants Capable of Releasing Drugs

Mesoporous surfaces can be provided to implants by combining mesoporous TiO_2_ with active hydrophobic or hydrophilic low molecular weight substances that promote osseointegration by providing nano-topography on the surface. A film has been developed that contains 6 nm-sized pores and 200 nm-thick TiO_2_ mesoporous film that is coated on titanium implants. These films were previously loaded with drugs used for osteoporosis (Raloxifene and Alendronate drugs). De novo bone growth and fixation of the implant was observed in implants with and without the drugs. The Raloxifene drug demonstrated significantly more favorable results, while improved bone implant fixation was revealed with both the drugs [226]. Similarly, these mesoporous films can be used to load antibacterial agents and elements that help in the metabolism and proliferation of osteoblast cells in the body [227,228,229]. Titanium coating enhances the chemical and mechanical properties and biocompatibility of polyetheretherketone (PEEK) implants without beam evaporation.

##### The TiO_2_-Based Biosensors

Nano-sized semiconductors have electrodes made out of TiO_2_ due to their large surface area, exceptional biocompatibility, and a porous structure. TiO_2_ may be used as an immobilizing matrix that reacts with carboxyl and amine groups in enzymes and helps them in retaining the biocatalytic activity [230,231]. The TiO_2_-based nanocomposites have gained interest for use in biosensors. Chitosan, with nanowires of TiO_2_ and graphene oxide that have been electro-reduced and are a part of a DNA-functionalized nanocomposite, has been utilized for detecting gene sequences in Vibrio parahaemolyticus. The interface area in nanocomposites can be enhanced due to TiO_2_ nanowires [232]. Various biomarkers, such as proteins, metabolites, DNA, lipids, RNA, etc., can be detected with TiO_2_-based nano-biosensors [233]. The analytical performance and desirable sensitivity of TiO_2_-based PEC biosensors have acquired interest in recent times [234]. However, the higher sensitivity and selectively functioning of TiO_2_-based nano-biosensors are required through advanced research for these biosensors to be incorporated in a clinical environment [235].

##### The TiO_2_ NPs in Sunscreens

The TiO_2_ NPs are excessively valuable NPs because of their potent role against the spread of various skin cancers occurring as a result of extra exposure to ultraviolet radiation [236,237]. These NPs are capable of effectively scattering the UV photons, which increases sun protection factor (SPF-1) in sun creams and thus prevents the photo damage [238,239,240]. Additionally, this photo-protective capability of TiO_2_ NPs provides aliments in other skin diseases as well [241,242]. The United States Food and Drug Administration (FDA) has declared the TiO_2_ NP-induced sunscreen safe [243] and gave it the commercial name of Eusolex-^®^-2000 [244]. These sunscreens are transparent and give brightness to the skin and are thus approved by the users [245,246]. A study concluded that TiO_2_ NPs of 62.1 nm in diameter showed the maximum protection against skin cancer and other skin diseases [247]. The European Commission’s Scientific Committee on Consumer Safety (SCCSS) declared that 25% TiO_2_ NPs in the sunscreen is safe with no risk of cytotoxicity when applied on sunburned skin or healthy intact skin [248,249]. Various other researchers also supported the idea that the inclusion of TiO_2_ NPs in sunscreen lotions is totally safe and does not penetrate deeply in the skin and thus reduces the chance of any cytotoxic effect [250,251,252,253,254].

##### The TiO_2_ NPs in Anticoagulants and Wound Dressings

The significant current medical applications of TiO_2_ NPs include their utilization as anticoagulant [255] wound dressings and skin tissue engineering [256,257,258]. The anticoagulant properties of these NPs inhibit the blood clot formation in the heart and brain, thus preventing their damage [255].

#### 3.3.2. Significance of TiO_2_ NPs as a Dental Biomaterial

The dental applications of the TiO_2_ NPs are illustrated in the Figure 14b. 

##### TiO_2_ NPs in Orthodontics

Recently, TiO_2_ NPs have been introduced in dental composite resin adhesives utilized for bonding purposes in orthodontics for increasing mechanical strength and antimicrobial properties [230]. The different percentages of TiO_2_ NPs in conventional composite resin revealed excellent antibacterial activity against *S*. *sanguinis* and *S*. *mutans* with maximum shear bond strength to enamel in 5% by weight [259]. The TiO_2_ NPs are also used in the orthodontic wires due to the increased stability, durability, biocompatibility, and non-allergic potential [179,260,261]. The (TiO_2_) NPs were also used for treating the surfaces of orthodontic wires. It was detected by the researchers that orthodontic wires without surface coating with TiO_2_ NPs showed enhanced bacterial adhesion (*S*. *mutans*) in the oral cavity in comparison to surface orthodontic wires with a surface coating of TiO_2_ NPs [262]. In another study, it was concluded that ceramic orthodontic brackets with a surface coating of TiO_2_ NPs displayed maximum antimicrobial activity against pathogenic bacteria (*S*. *mutans* and *C*. *albicans*) [263]. This shows the potent role of TiO_2_ NPs in the field of orthodontics [264].

##### The TiO_2_ NPs in Dental Implants

Dental implants are commonly made of titanium due to its strong mechanical and chemical properties. The dental implants made of TiO_2_ NPs showed both enhanced osteogenic activity and antibacterial activity but still there are a few chances of peri-implantitis, which can be overwhelmed by modifying the surface of the TiO_2_ implant with other materials. The TiO_2_ NPs are the most versatile and compatible nanomaterials, which can be used in combination with many other elements without producing any toxic effect [265,266]. The surface modification of TiO_2_ implants with Zinc made it not only novel and gave it cytocompatibility but also increased its antibacterial functions against *S*. *mutans*, *Porphyromonas gingivalis* [267]. 

Moreover, the modification of the TiO_2_ implant with multilayers of TiO_2_ NPs enhanced its adhesion and strength in the bone [268]. The heat-treated TiO_2_ implants also revealed potent osteoblastic viability and antibacterial activity against *Porphyromonas gingivalis* [269]. The hydrothermally treated TiO_2_ implant exhibited non-cytotoxic behavior against mammalian cell lines and increased deposition of calcium from the osteoblast with antimicrobial activity against methicillin resistant *Staphylococcus aureus* [270]. Thus, it is essential to protect the adhesion of bacteria on implant surfaces in order to prolong their longevity, stability, and durability by preventing infections [271,272,273]. The additional property of TiO_2_ NPs is their strong biocompatibility with blood, which can easily allow their surface modifications in the blood with compatible implants [274]. The nerve regeneration via Schwann cells to dental implants occurs through HA nanocomposite ceramic coating to the TiO_2_ NPs [275]. Currently, novel, multilayered (TiO_2_) NP-based dental implants are being introduced for their utilization [276]. Due to the improved cell adhesion, proliferation, migration, differentiation, and high contrast wettability of TiO_2_ NP-based surfaces, these NPs have become a promising approach for yielding high strength scaffolds of implants [277,278,279]. In a study conducted previously, TiO_2_ NPs were grown as nanotubes on titanium surfaces and incorporated with ZnO NPs with a diameter of 20 to 50 nm and were bonded to the walls of the TiO_2_ nanotubes. These dominantly TiO_2_ NP-based implants provided strong antibacterial activity against *S*. *mutans* and *Porphyromonas gingivalis* through a constant and slow release of zinc ions [280]. In another study, TiO_2_ NPs were used as TiO_2_ nanorod arrays (TNFRs) to form surface modified implants. The TiO_2_ nano-array modified Ti substrate promoted the adhesion, proliferation, and osteogenic differentiation of human periodontal ligament stem cells. In addition, TiO_2_ nanorod arrays (TNRs) exhibited superior antifungal/antibacterial properties [281,282]. The TiO_2_ NP-based implants coated with the hydroxyapatite nanocomposites were also successful in providing the strong bone tissue osseointegration [283].

##### TiO_2_ NPs in Oral Disinfectants and Mouth Washes

Dental caries or tooth decay is a common but pathogenic oral disease associated with the dissolution of enamel and dentin surfaces of teeth because of acid-producing bacteria. This in turn leads to white spot fabrication initially and followed finally by tooth loss. This global problem of the tooth decaying process can be easily avoided by utilizing the latest oral disinfectants with metal oxide NPs, as compared to chlorhexidine, which is a conventional oral disinfectant [284]. Previously published research depicted the potent antimicrobial activity of TiO_2_ NP-containing oral disinfectants against *Streptococcus mutants*, which are the pathogenic species in the oral cavity responsible for the tooth decay or dental caries [285]. Additionally, these TiO_2_ NP-containing oral disinfectants and mouthwashes are helpful in preventing the formation of white spots on the teeth surfaces after the orthodontic alignment [264].

##### TiO_2_ NPs in Restorative Materials

This success of the restorative material in dentistry is greatly dependent on its biological strength and chemical, mechanical, and physical properties [286]. The wide range applications of TiO_2_ NPs in bases, composites, sealants, cements, adhesives, and lining materials displayed immensely effective antimicrobial activity. Previously, Garcia-Contreras et al. [79,287,288], revealed that TiO_2_ NP-induced glass ionomer cement is a novel restorative material introduced in dentistry as a result of its longevity, stability, and antimicrobial activity against strong masticatory loads. In another study, a novel TiO_2_ NP-based GIC restorative material proved to have a long shelf life with strong antimicrobial activity against *S*. *mutans* [272]. The addition of TiO_2_ NPs in conventional composite restoration gave rise to the latest nanocomposites with cytocompatibility and antimicrobial activity against *E*. *coli* and *S*. *aureus*, which are also responsible for the origin of tooth decay or dental caries [271]. Moreover, Ag doped with TiO_2_ NPs has been also used as a restorative material due to its strong antimicrobial activity against *S*. *aureus* [289]. The addition of different concentrations of TiO_2_ NPs in GIC restorative materials has a great impact on mechanical and physicochemical properties. In a study, TiO_2_ NPs with a mean particle size of about 21 nm have been incorporated into GIC restorative material to investigate its mechanical and physicochemical properties. In the presence of TiO_2_ NPs, the flexural strength, hardness strength, compressive strength, enamel shear bond strength, and dentin shear bond strength of the novel titanium oxide NP-based GIC significantly increased in comparison to the conventional GIC restorative material [290]. In another study, the incorporation of a combination of 2 wt% TiO_2_ NPs and 1 wt% of CnC in conventional GIC restorative material enhanced the compressive strength by 18.9% and the tooth enamel shear bond strength up to 151% when compared to the conventional GIC restorative material [291].

The TiO_2,_ ZnO, and Ag NPs were incorporated in conventional GIC 10%wt and were tested for their mechanical and physicochemical properties. The TiO_2_ NPs used in this study had pores and thus, they were stronger than ZnO NPs and Ag NPs. The TiO_2_ NPs and ZnO NPs had significantly higher compressive strength while the compressive strength of the Ag NPs was equal to the control group of the conventional GIC restorative material [292]. The addition of 3% (*w*/*w*) TiO_2_ NPs in the GIC restorative material improved the mechanical and antibacterial properties without interfering with the release of the fluoride capacity of GIC, which is an advantage of TiO_2_ NP-induced GIC [293]. The addition of TiO_2_ NPs significantly improved the mechanical and antibacterial activity in different previous research performed by Elaska et al. and J. Sun et al., but only up to 3% [291,294]. 

Recently, research conducted by A. Mansoor et al. [79,295] confirmed that the addition of 3 and 5 wt% TiO_2_ NPs into the conventional GIC significantly increased its flexural strength and compressive strength as compared to the conventional GIC.

##### The TiO_2_ NPs in Whitening Agents

A TiO_2_ nanoparticle has displayed increased aesthetics in terms of whitening in dentistry. In a study, TiO_2_ NPs were added into 3.5% of a H_2_O_2_ whitening agent that enhanced the aesthetics of the enamel by improving its color, as compared to the conventional 3.5% of H_2_O_2_ whitening agent without TiO_2_ NPs [296,297]. The TiO_2_ NPs with a doping of nitrogen have revealed antimicrobial activity with an increased whitening effect and reduced sensitivity [298]. In addition, the mixture of TiO_2_/Ag NPs in H_2_O_2_ was used as a whitening agent and then tested for its cytotoxicity through in vivo testing. The mixture of TiO_2_/Ag NPs showed lesser cytotoxicity when compared to the whitening agent containing only H_2_O_2_ [299].

In a previous study, TiO_2_ NPs were mixed with H_2_O_2_ and were applied in a gel to increase the whitening efficacy of hydrogen peroxide (H_2_O_2_). Thus, in the presence of TiO_2_ NPs, the H_2_O_2_ concentration could be decreased to 6% with no clinical difference in the whitening effect compared with the H_2_O_2_ of 35%, but tooth sensitivity was reduced when the H_2_O_2_ concentration was reduced to 6% and the TiO_2_ NPs used [300].

##### The (TiO_2_) NPs in Acrylic Resins

The poly-methyl-methacrylate (PMMA) is the best denture base resin material used for the construction of dental prosthesis, such as complete and partial dentures. This PMMA is recommended because of its biocompatibility, biosafety, aesthetics, low weight, and low cost in dental prosthesis [301]. The physical, chemical, and mechanical properties of the dental prosthesis based on PMMA are reduced, which results in defects, including voids, irregularities, pores, and cracks [302]. These defects reduce the mechanical properties [303] and increase the growth and multiplication of microorganisms on these prosthesis [304,305]. The most pathogenic microorganism that gets attached to this dental prosthesis is *Candida albicans*, which can cause severe infections in the oral cavity if not controlled [306,307]. The properties of PMMA can only be improved with the incorporation of nanotechnology [308,309,310].

The (TiO_2_) NPs are also introduced into the PMMA of acrylic resins in order to increase their mechanical strength and hardness. Furthermore, the addition of these NPs revealed superior photopolymerization, biocompatibility with low levels of cytotoxicity, mutagenicity, and genotoxicity [311]. The binding effect of TiO_2_ NPs in the PMMA of acrylic resins has been immensely enhanced for the dental prosthesis [312]. This became possible due to a strong bond formation between the carbonyl group of PMMA and the hydroxyl group of the (TiO_2_) NPs, resulting in the increased physical, chemical, and mechanical properties of these prostheses [313,314]. In addition, it also reduces the chance of the growth and proliferation of various pathogenic microorganisms, especially Candida albicans, due to the increased antimicrobial activity obtained with the addition of TiO_2_ NPs [315,316]. The TiO_2_ NPs are considered to be the best possible option to be added in PMMA because of their low investigated toxicity on living cells as compared to another NPs [317,318,319,320]. 

The TiO_2_ NPs have a large spectrum of activity against microorganisms, including both Gram-negative and Gram-positive bacteria and fungi [312]. Previously, when the ratio of TiO_2_ NPs was increased in PMMA, it further improved the antimicrobial behavior of PMMA by significantly reducing the bacterial adherence [311]. The PMMA incorporation with the mixture of the nanofillers of TiO_2_ and silicon dioxide mixture had a superior antibacterial activity under UV, which could degrade microorganisms with prolonged exposure. This study concluded that TiO_2_ NPs are compatible and safe when used in combination with other elements, metals, and alloys for dental purposes [321].

##### The TiO_2_ NPs in Oral Dentifrices

The TiO_2_ NPs are also incorporated in the oral dentifrices in 1–10% by weight for prophylaxis of sensitivity and caries [322]. These NPs are employed in oral dentifrices in the mean particle sizes ranging between 100 and 300 nm, which are easily soluble in enamel, dentine, and cementum and thus prohibit the onset of caries and tooth sensitivity [323]. The strong binding of organic molecules in dental tissues (enamel) to TiO_2_ NPs allows the release of a small amount fluoride from oral dentifrices which gets trapped in a tooth’s matrix. Eventually, the matrix of the tooth becomes the least soluble with the minimal fluoride release over time [324]. In addition, the previous literature also supports the idea that the increased binding of TiO_2_ NPs to dental tissues (enamel) reduces the size of hydroxyapatite crystals of enamel, resulting in advanced hue, value, and chroma, which maintains the color of human tooth enamel [325,326,327]. 

##### The TiO_2_ NPs in Dental Adhesives

The adhesives are the restorative materials used in dentistry with minimal intervention [328]. The most commonly used adhesives in dentistry are dentin adhesives [329]. The bond formed between the dentin adhesive and the dentin surface becomes unstable and gets loose over time [330]. This occurs due to the collagenous, hydrophilic nature of the dentin structure and the breakage of the strong polymeric covalent bond between them through hydrolytic degradation. Eventually, the total failure of the adhesive bond to the dentin surface occurs [331]. The incorporation of TiO_2_ NPs in adhesives to investigate their bonding to human teeth has been carried out [332].

Recently, research incorporating TiO_2_ NPs in dental adhesives concluded that they had enhanced biocompatibility with reduced solubility and water sorption when compared to the conventional adhesives used in dentistry. Thus, the usage of these NPs is highly recommended in currently available adhesive dentistry [333]. Another research work elaborated that TiO_2_ NPs added to self-adhesive cements revealed a maximum degree of conversion (DC) when compared to their conventional types [334]. The TiO_2_ NPs are also capable of improving the mechanical and physicochemical properties of adhesives when used as fillers [335]. Thus, TiO_2_ NPs are potent enough to be deployed as reinforcing fillers in dental adhesives due to their multiple advantages [336,337].

Previously, TiO_2_ NPs were mixed with a light-curable orthodontic composite paste to form TiO_2_ NP-based resin composite adhesives which were able to reduce enamel demineralization and provide antibacterial properties for dental adhesive systems. The shear bond strength and the adhesive remnant index for the nanocomposites with TiO_2_ NPs were similar to those of the control composite [259]. In another study, nanocarbons containing TiO_2_ NPs were added together to form biomineralized adhesive material in dentistry. The photocatalytic activity of this newly formed nano-adhesive incorporated with TiO_2_ proved to interfere with the bacterial acidity and was found to have a positive effect on tooth re-mineralization in simulated body fluids [338]. 

##### The TiO_2_ NPs in Dental Prosthesis (Veneers, Crowns, and Bridges)

The durability and appearance of the tooth is improved by replacing upper enamel layers with artificial materials such as sapphire, diamond, and ceramics because of their good biocompatibility and aesthetics. These materials are brittle and prone to fracture; therefore, NPs are added to improve their mechanical properties [339]. 

Currently, TiO_2_ NPs are incorporated in ceramics to enhance the aesthetics and mechanical properties of crowns and bridges. This addition of TiO_2_ NPs in ceramics not only enhanced their aesthetics but also their mechanical properties when compared to the conventional ceramics without TiO_2_ NPs [179,260,261,340]. This became possible due to the pure white appearance, fatigue resistance, light weight, low elastic modulus, biocompatibility, corrosion resistance, and strength [180]. 

Titanium is a naturally available common and pure element that shows low toxicity and no allergic reactions when compared to other metals and elements used for dental prosthesis, including cobalt, beryllium, nickel, iron, steel, stainless steel, chromium, etc. Thus, TiO_2_ NPs are considered as biosafe, biocompatible, easily available, and cost-effective nanomaterials to be utilized in dental prosthesis such as veneers, crowns, and bridges used in the oral cavity [48,49,341]. 

##### The TiO_2_ NPs in Scaffolds/Bone Grafting of Maxillofacial Regions

Naturally, bone is a nano-scaled material which is less than 100 nm in dimension. The materials used for bone grafts in the maxillo-facial regions, including the face, oral cavity, head, neck, ear, and nose, are composites composed mainly of organic compounds (mainly collagen) reinforced with inorganic ones. The nano-crystallites show a loose microstructure, with nanopores situated between the crystallites. This material structure will be completed by filling the pores in the micrometer area. By following this process, a rough surface area is formed on the boundary layer between the biomaterial and the cell, which is very important for fast cell growth. Special features that should be included in bone graft materials are osteoinduction, good processibility, non-sinter, synthetic, highly porous, nanostructure form, and ability to undergo degradation by osteoclasts [342,343].

The TiO_2_ NPs can be used in bone grafting because of the increased strength, biocompatibility, low modulus of elasticity, corrosion resistant nature, and the extremely light weight of titanium used in the graft composition as compared to other elements and metals [180]. All these characteristics make titanium an ideal biomaterial, especially for its insertion in the human body in the form of metal prosthesis. Moreover, titanium is the choice of material for making internal metal plates, wires, and meshes, such as fracture fixations, cranioplasty plates, cranioplasty meshes, cranioplasty wires, and coronary artery stents [344,345,346]. Additionally, titanium meshes and scaffolds, as well as prosthesis, are well known for nose, eye, maxilla, mandible, ear reconstruction procedures, and ossicular replacements [261,340,347]. 

##### The TiO_2_ NPs in Endodontics

The titanium metal and its metal oxide alloy, commonly known as titanium oxide, are utilized for making the maxillary and mandibular obturators, files, spreaders carvers, and reamers [260,261]. The various dental instruments made from a combination of nickel and titanium are considered ideal for endodontic procedures as a result of their flexibility that makes them fracture resistant, e.g., files, spreaders, carvers, etc. [348,349,350].

### 3.4. The TiO_2_ NPs: The Most Biocompatible Material for Medical and Dental Applications

The ideal oral biomaterial should exhibit good thermal, optical, electrical, biological, mechanical, physical, and chemical properties, along with an outstanding biocompatibility [353]. The TiO_2_ NPs possess almost all the properties of an ideal oral biomaterial; therefore, these NPs are used as antimicrobial agents, dental restorative material, dental prosthetics; they reduce bacterial adhesion to the tooth surface and provide protection against dental caries, bleaching agents, dental implants, and orthodontics. These TiO_2_ NPs revealed a long-term effect on dental implants; the surface modification provided more advantages such as less bacterial adhesion, improved hardness, biocompatible, low toxic effect, chemical stability, low density, significant adsorption ability; most importantly, they are cost-effective and have strong antimicrobial activity and enhanced mechanical properties. They also displayed extraordinary functionality, long-term stability, durability, fatigue resistance, high mechanical resistance, low thermal diffusivity, extremely light weight, corrosion and wear resistance, high electrical conductivity, and low thermal conductivity, thus enhancing their stability, sustainability, and longevity. They became the most versatile, advanced, and adaptable nanomaterials as a result of their immensely increased mechanical strength, hardness, lightness, and white color. The TiO_2_ NPs display a large spectrum of antimicrobial, antibacterial, antibacterial, antibactericidal, antiparasitic, and antifungal activity against various pathogenic microorganisms, including Gram-negative bacteria, parasites, Gram-positive bacteria, and fungi. The TiO_2_ NPs are nontoxic, and the American Food and Drug Administration (FDA) has approved TiO_2_ for use in human food, drugs, cosmetics, and food contact materials [354,355,356,357,358,359]. Previous research [48,49,115,179,312,360,361,362,363,364,365,366,367,368,369,370] in the context of biosafety and biocompatibility concluded no cytotoxic effect of different concentrations (1–20) mg/cm^2^ after oral administration. The larges dose of 1000 mg/kg BW/day did not produce any toxic effect or allergic reactions on encountering TiO_2_. Several studies had reported that the intake of different doses of TiO_2_ NPs/Kg body weight did not lead to considerable capturing of TiO_2_ from GIT into the blood or different internal organs, and it was also found that absorption from GIT into blood, urine, and other organs was scarce.

## 4. Conclusions and Futures Prospects

The TiO_2_ NPs are safe enough to be used in advanced medical and dental applications as a result of their biocompatibility, biosafety, and non-allergic reactions with the human tissues. Until today, size, shape, phase form, surface morphology, coating, and topography were considered as the significant features responsible for their biocompatibility. Current research on TiO_2_ NPs has revealed that the mode of synthesis is the most important factor responsible for the biocompatible nature of these NPs, favoring their induction in medical and dental applications. This is due to the fact that the synthesis route can be controlled by temperature, pressure, pH, melting point, environment, and stability of solution medium utilized during the synthesis of these NPs. Moreover, these TiO_2_ NPs are cost effective and easily available and that became the sole reason for their employment. Therefore, future studies should incorporate the most suitable synthesis mode in order to produce biosafe, biocompatible and ecofriendly TiO_2_ NPs for utilization the in latest nanomedicine and nanodentistry. 

## Figures and Tables

**Table 1:** Comparative differences between conventional and biological TiO_2_ NPs [40,51,85,90,91].

Properties	Conventional TiO_2_	Biological TiO_2_	References
Nature	Expensive, toxic	Cost effective, nontoxic	[40]
Reducing agent	Dimethylformamide, ethylene glycol, hydrazine hydrate, sodium borohydride, polyol, sodium citrate, and N,N-dimethylformamide	Biomolecules include phenolics, polysaccharides, flavones, terpenoids, alkaloids, proteins, amino acids, enzymes, predominantly, and nitrate reductase	[51]
Method	Stabilizer (surfactant) is added to the first solution to prevent the agglomeration of NPs	There is no need to add a stabilizing agent	[85]
Environmental impact	Environmental pollution is a disadvantage of the chemical method and the chemical reduction methods are energy-intensive	Synthesis carried out in environmental conditions, and they are safe enough and consume no energy	[90]
Antibacterial activity	The chemically synthesized NPs show comparatively lower antimicrobial activity against pathogenic bacteria	The NPs synthesized from biological means are showing better antimicrobial activity against the pathogenic bacteria	[91]
Stability	NPs are not stable	NPs are highly stable	[85]
Byproducts formation	Harmful byproducts formed	No harmful byproducts formed	[91]
Biocompatible coating formation	No biocompatible coating is formed	The additional safe and effective surface area for interaction in the biological environment is produced due to the biocompatible coating on the nanoparticle’s surface	[51]
Process	Non-economical	Economical	[40]

## Data Availability

Not applicable.
